# Reducing bias in microbiome research: Comparing methods from sample collection to sequencing

**DOI:** 10.3389/fmicb.2023.1094800

**Published:** 2023-03-30

**Authors:** Jolanda Kool, Liza Tymchenko, Sudarshan A. Shetty, Susana Fuentes

**Affiliations:** ^1^Centre for Infectious Disease Control, National Institute for Public Health and the Environment (RIVM), Bilthoven, Netherlands; ^2^Department of Medical Microbiology and Infection Prevention, Virology and Immunology Research Group, University Medical Center Groningen, Groningen, Netherlands

**Keywords:** human studies, microbiota, reproducible analysis, microbiome, gut, 16S rRNA gene sequencing

## Abstract

**Background:**

Microbiota profiles are strongly influenced by many technical aspects that impact the ability of researchers to compare results. To investigate and identify potential biases introduced by technical variations, we compared several approaches throughout the entire workflow of a microbiome study, from sample collection to sequencing, using commercially available mock communities (from bacterial strains as well as from DNA) and multiple human fecal samples, including a large set of positive controls created as a random mix of several participant samples.

**Methods:**

Human fecal material was sampled, and aliquots were used to test two commercially available stabilization solutions (OMNIgene·GUT and Zymo Research) in comparison to samples frozen immediately upon collection. In addition, the methodology for DNA extraction, input of DNA, or the number of PCR cycles were analyzed. Furthermore, to investigate the potential batch effects in DNA extraction, sequencing, and barcoding, we included 139 positive controls.

**Results:**

Samples preserved in both the stabilization buffers limited the overgrowth of Enterobacteriaceae when compared to unpreserved samples stored at room temperature (RT). These stabilized samples stored at RT were different from immediately frozen samples, where the relative abundance of Bacteroidota was higher and Actinobacteriota and Firmicutes were lower. As reported previously, the method used for cell disruption was a major contributor to variation in microbiota composition. In addition, a high number of cycles during PCR lead to an increase in contaminants detected in the negative controls. The DNA extraction had a significant impact on the microbial composition, also observed with the use of different Illumina barcodes during library preparation and sequencing, while no batch effect was observed in replicate runs.

**Conclusion:**

Our study reaffirms the importance of the mechanical cell disruption method and immediate frozen storage as critical aspects in fecal microbiota studies. A comparison of storage conditions revealed that the bias was limited in RT samples preserved in stabilization systems, and these may be a suitable compromise when logistics are challenging due to the size or location of a study. Moreover, to reduce the effect of contaminants in fecal microbiota profiling studies, we suggest the use of ~125 pg input DNA and 25 PCR cycles as optimal parameters during library preparation.

## Introduction

Over the last decades, numerous studies have shown the value of investigating the role of the human microbiome in health and disease. The intestinal microbiome has been associated with several disorders, ranging from gastrointestinal diseases such as inflammatory bowel disease or colorectal cancer (Halfvarson et al., 2017; Johns and Petrelli, 2021) to systemic disorders such as obesity and diabetes (Turnbaugh et al., 2009; Requena and Velasco, 2019; Fan and Pedersen, 2021). The intestinal microbiome interacts with the host immune system (Honda and Littman, 2016; Thaiss et al., 2016) and has been shown to play a role in the response mounted to vaccines, such as vaccine-induced gut mucosal antibody response to the oral polio vaccine (OPV) (Zhao et al., 2020) and the rotavirus vaccine (RVV) (Harris, 2018). In recent years, technological advances in next-generation sequencing (NGS) have made it more accessible to study the human microbiome. With a reduction in sequencing costs and an increase in capacity for high-throughput sequencing, the number of studies that investigate the human microbiome has expanded dramatically. Although this increase in available data and knowledge is beneficial for the field, the urge to ensure reproducible and comparable results has become one of the most important challenges facing researchers nowadays.

Several studies compare different methods for microbiome research through the entire workflow, from sample collection to sequencing approaches (Costea et al., 2017; Tourlousse et al., 2021). Recently, the STORMS checklist was published providing a guideline for concise and complete reporting of microbiome studies (Mirzayi et al., 2021). For gut microbiome research, the most widely accepted method for sampling fecal samples is freezing upon collection, in which participants are usually asked to collect their fecal sample at home and store it in a home freezer before transportation to the laboratory for further processing (Wu G. D. et al., 2010; Bahl et al., 2012; Fouhy et al., 2015). While optimal for sample storage, this method is often logistically difficult and expensive, becoming a limiting factor when conducting large-scale studies, especially in regions where the maintenance of the cold chain during transportation is challenging. Therefore, alternative sampling methods are essential to facilitate large population-level microbiome studies from broad geographic locations. To this end, several approaches have been tested aiming to stabilize the microbial composition in fecal samples during transportation at ambient temperatures, such as the OMNIgene·GUT system, the Stratec stool collection tube, or the Stool Nucleic Acid Collection and Preservation Tube (Chen et al., 2020; Lim et al., 2020; Jones et al., 2021).

Furthermore, after sampling, the extraction of nucleic acids is shown to be a critical step affecting the outcome of microbiome studies. Decisions in approaches to break the cell wall, through chemical lysis or mechanical disruption (bead-beating), or even the composition and size of beads used, can significantly impact the composition of the fecal microbiome (Salonen et al., 2010; Yang et al., 2020). After DNA is extracted, several other factors such as PCR conditions (Hasrat et al., 2021), choice of primers, and downstream bioinformatics approaches can impact the end results of fecal microbiome analyses (O'Sullivan et al., 2021; Nearing et al., 2022; Szóstak et al., 2022).

In our study, we aimed to contribute to the existing knowledge and work toward a controlled and reproducible wet-lab workflow for the study of the fecal microbiome. To that end, we examined the effect of different sample collections and short-term storage conditions, using the OMNIgene·GUT and Zymo research stabilization systems. In addition, we studied the effect of cell disruption using chemical or mechanical methods and tested different DNA purification kits. Finally, we investigated the impact of bacterial input and the number of PCR cycles during library amplification, and analyzed the inter- and intra-run variations in large-scale studies, by testing the effect of DNA extraction rounds and the use of different barcodes on the overall microbial composition.

## Materials and methods

### Sample selection and study design

Samples were selected from the PIENTER3 and Z-test study (Table 1). PIENTER is a cross-sectional study, designed to periodically monitor the seroprevalence of National Immunization Program (NIP)-targeted diseases in the Netherlands. During the third survey in 2016–2017 (PIENTER3), fecal samples were collected from participants throughout the Netherlands and the Caribbean islands Bonaire, St. Eustatius, and Saba (Verberk et al., 2019). The *Z*-test study was designed for protocol optimization. None of the participants of both studies had undergone antibiotic treatment 3 months prior to collection, and informed consent was obtained from all participants.

**Table 1:** Demographics of the study participants.

	**PIENTER3 (*n* = 64)**	***Z*-test (*n* = 12)**
**Gender, (** ***n*** **, %)**		
Male	23 (32.9)	8 (66.7)
Female	47 (67.1)	4 (33.3)
**Age**		
Mean	41.3	38.1
SD	24.6	7.7
Range	0–82	24–51

**Figure 1:**
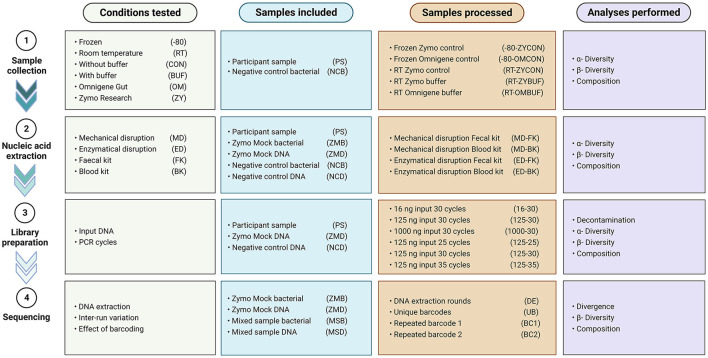
Overview of the conditions tested, samples included and processed in every experiment, and analyses performed.

To investigate the effect of several aspects known to impact a microbiome study [e.g., sample collection, nucleic acid extraction, library preparation, and sequencing through different approaches (Figure 1)], we compared different approaches, and results were analyzed using 16S rRNA gene amplicon sequencing data.

### Sample collection

For sample collection under standard conditions, participants were asked to freeze their fecal samples in their home freezers (~-20°C) directly upon collection. Subsequently, samples were transported to the laboratory on dry ice and stored at −80°C until further processing. Within the PIENTER3 study, a subset of participants (*n* = 64) collected samples, and two aliquots were taken which were subsequently treated separately as described later. Next to the standard condition, samples were collected in the OMNIgene·GUT tube (DNA Genotek, Ottawa, Canada) containing 2 mL of stabilization buffer and stored at room temperature (RT) for 3–5 days before freezing (−80°C). For the *Z*-test study, all participants (*n* = 12) collected samples and stored three aliquots of the material. The first sample was collected under standard conditions, the second sample was stored at RT for 3–5 days, and the third sample was collected in a Zymo research tube (Zymo Research Inc., Irvine, CA, USA) containing 9 ml of DNA/RNA shield and stored at RT for 3–5 days before storage at −80°C. A positive control (mixed sample-bacterial or “MSB”) was generated by thoroughly mixing equal amounts of fecal material from five randomly selected healthy participants from the PIENTER3 study. This mix was evenly aliquoted and stored at −80°C until further use in different random DNA extraction runs (*n* = 23). DNA extracted from these aliquots (mixed sample DNA or “MSD”) was used as a control in different library preparations (*n* = 12). In addition, microbial community standards (ZymoBIOMICS, Zymo Research, Irvine, CA, USA) were used as positive controls. We used the ZymoBIOMICS Microbial Community Standard as the control for DNA extractions (ZMB) and the ZymoBIOMICS Microbial Community DNA Standard as control during library preparation (ZMD). Negative controls were included in every DNA extraction, where the appropriate buffer was used depending on the collection method, without adding any fecal material. DNase-free water was used as a negative control during library preparation.

### DNA extraction

DNA was extracted from fecal material by the mechanical disruption of the cells, and a subset of samples was in parallel treated *via* enzymatic lysis. To lyse the cells enzymatically, tubes containing 0.25 g of fecal material, 1 mL of lysis buffer (Promega, Madison, USA), and 40 μl of Proteinase K (Promega, Madison, USA) were vortexed and heated at 95°C for 5 min. After heating, the samples were cooled down and incubated at 56°C for 5 min. Samples were centrifuged (17,000×g for 5 min) and the supernatant lysates were collected for further processing. For mechanical disruption, we used a repeated bead-beating approach, using pre-assembled tubes containing 0.5 g zirconia/silica beads (0.1 mm) and five glass beads (2.7 mm) (BioSpec Products, Bartlesville, OK, USA). In tubes without stabilization buffer, 0.25 g of fecal material and 700 μl of S.T.A.R. buffer were added to the beads. For fecal material collected in the Zymo research tubes, 1 mL of the stabilization buffer containing the fecal sample was added to the beads. From the material collected in the OMNIgene·GUT tubes, 250 μl of the stabilization buffer and 450 μl of S.T.A.R. buffer were added to the beads. All samples were thawed at RT and lysed by repeated bead-beating (5.5 ms for 1 min, repeated three times, and cooled on ice for 5 min) in a Fastprep-24 (MP Biomedicals, Irvine, USA), followed by heating of the samples at 95°C for 15 min. Samples were centrifuged and the lysates were collected. All lysates were further purified in the Maxwell RSC instrument (Promega, Madison, USA), using the Maxwell RSC blood DNA kit and the Maxwell RSC Fecal kit on a subset of the samples. DNA was eluted in 60 μl of elution buffer and further purified by using the OneStep PCR Inhibitor Removal Kit (ZymoBIOMICS, Zymo Research, Irvine, CA, USA). A total of 48 samples were processed in every DNA extraction round, including two negative and two positive controls.

### Quantification of bacterial DNA by quantitative PCR

DNA concentration was measured using a Quantus Fluorometer (Promega, Madison, USA), and samples were stored at −20°C until further processing. The bacterial load present in the purified samples was measured by quantitative PCR (StepOnePlus Real-Time PCR System, Thermo Fisher Scientific, the Netherlands), using a universal primer set targeting the 16S rRNA gene (forward Eub341F: CCTACGGGAGGCAGCAG, reverse Eub534R: ATTACCGCGGCTGCTGGC) (Muyzer et al., 1993). The quantitative PCR was carried out by using SYBR Green, in a 25 μl reaction consisting of 12.5 μl of Maxima SYBR Green/ROX qPCR Master Mix (Thermo Fisher Scientific, Waltham, MA, USA), 0.5 μM forward primer Eub341F, 0.5 μM reverse primer Eub534R, 2 μl of DNA (500 times diluted in HPLC grade water), and 8 μl of HPLC grade water. The DNA was denaturated (95°C; 10 min), followed by 40 cycles of denaturation (95°C; 15 s), annealing (60°C; 15 s), extension (72°C; 15 s), and a holding stage (95°C; 1 min and 60°C; 1 min).

### Library preparation and sequencing of the V4 region of the 16S rRNA gene

Results obtained from the qPCR were used to equalize the number of bacteria present in all samples and provide an input of 100 pg DNA for the amplification of the hypervariable V4 region of the 16S rRNA gene, using the 515F (5′-GTG CCA GCM GCC GCG GTA A-3′) and 806R (5′-GGA CTA CHV GGG TWT CTA AT-3′) primers, including the Illumina flow cell adapter and a unique 8-nt index key (Caporaso et al., 2011; Kozich et al., 2013; Thompson et al., 2017). The amplification mix consisted of 0.5 μl of (1 U) Phusion Hot Start II High-Fidelity DNA Polymerase, 5 μl of 5× Phusion HF Buffer (Thermo Fisher Scientific), 7 μl of HPLC grade water, 2.5 μl of 2 mM dNTP mix (Thermo Fisher Scientific, Waltham, MA, USA), 0.5 μM of forward primer 515F, 0.5 μM of reverse primer 806R, and 5 μl of template DNA. After denaturation (98°C; 30 s), 30 cycles were performed consisting of denaturation (98°C; 10 s), annealing (55°C; 30 s), extension (72°C; 30 s), and a final hold (72 °C; 30 s). For the optimization of the V4 amplicon PCR, the effect on the microbiota profile using a different amount of input DNA (16, 125, and 1,000 pg) and the number of cycles (25, 30, and 35 cycles) were investigated. The amplified product was checked on size and quantified to pool equimolar, using the QIAxcel DNA High-Resolution Kit on the QIAxcel Advanced System (Qiagen, Hilden, Germany). The pool was purified two times by 0.9× AMPure XP magnetic beads (Beckman Coulter, the Netherlands). The final quantification of the pool was done using the KAPA library quantification kit (Roche, USA). Paired-end sequencing was conducted using a V3 MiSeq reagent kit (600 cycles) on an Illumina MiSeq instrument (Illumina Inc., San Diego, CA, USA). The raw sequencing data are deposited at The European Nucleotide Archive (ENA) under the study accessionPRJEB59099.

### Processing of 16S rRNA gene sequence data

Sequence data were demultiplexed based on sample-specific barcode combinations and the primers were removed, prior to processing the raw reads using the DADA2 pipeline. Default parameters were used unless otherwise stated (Callahan et al., 2016). Reads were trimmed at 220 and 100 nt for forward and reverse reads, respectively, and filtered by truncating reads of a quality score ≤5. For inferring sequence variants, a minimum of 1,000,000,000 bases were used for error rate learning. The resulting amplicon sequence variants (ASVs) were cleared from chimera, and taxonomy was assigned with the RDP classifier and SILVA database (version 138.1) (Wang et al., 2007; Quast et al., 2013).

### Statistical analysis

Analyses were performed in R version 4.1.0 (R Core Team, 2022) using the microbiome package (Lahti and Shetty, 2017) among others. Alpha-diversity indices and beta-diversity ordinations (of Bray–Curtis distance) were calculated with the “phyloseq” package (McMurdie and Holmes, 2013). Smaller datasets (*n* = 76) were visualized with non-metric multidimensional scaling (NMDS), while PCoA was used for larger datasets. The Wilcoxon test from the rstatix package was used for pairwise comparisons and corrected for multiple testing using the Bonferroni method where appropriate. Community level differences in beta-diversity were tested using PERMANOVA for multiple comparisons from the ecole (v0.9-2021) R package, using 999 permutations and the Bonferroni method to correct for multiple testing (Smith, 2021). The heterogeneity of the microbiota was quantified using the divergence function of the microbiome package, by calculating the dissimilarity of each sample from the group mean. The significance of the divergence between all conditions was tested with the Wilcoxon test.

Linear discriminant analysis effect size [LEfSe (Segata et al., 2011)] was performed using LEfSe [Galaxy (harvard.edu)] with an alpha-value for the factorial Kruskal–Wallis test among classes of <0.05 and an LDA threshold of >4.0. Results were visualized in a cladogram. Identification of contaminant ASVs was done with the decontam (v1.8.0) R package using the prevalence method (threshold = 0.1) (Davis et al., 2018). Correlations of the Mock samples compared to the theoretical composition were calculated with Spearman's correlation using the “checkZymoBiomics” function from the chkMocks (v 0.1.03) package (Anand, 2022).

## Results

### Effect of sample collection on the overall diversity and composition of the fecal microbiome

**Figure 2:**
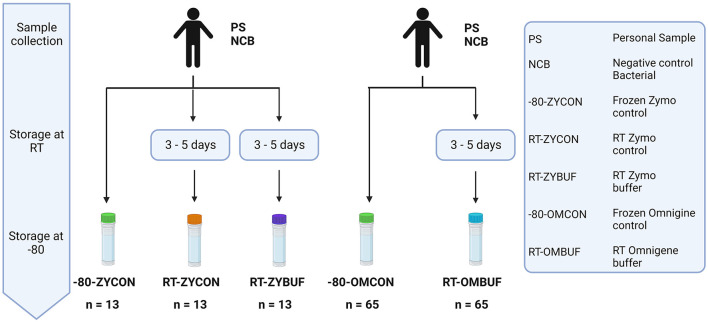
Overview of the different methods for sample collection and storage. The effect of storage at RT for 3–5 days was tested with and without stabilization buffer of the Zymo research (ZYBUF) and OMNIgene·GUT (OMBUF) collection tubes and compared to the standard storage condition (−80). Three aliquots of fecal material from 12 participant samples (PSs) and one negative bacterial control (NCB) were tested: one control sample stored under standard storage conditions (−80-ZYCON) and two additional copies stored at room temperature for 3–5 days prior to freezing at −80°C, with and without the Zymo Research stabilization buffer (named RT-ZYBUF and RT-ZYCON, respectively). In addition, fecal samples of 64 participants (PS) and one negative bacterial control (NCB) were analyzed under two conditions. In addition to the control sample collected under standard conditions (−80-OMCON), an additional aliquot was collected in the OMNIgene·GUT tube with a stabilization buffer and stored at RT for 3–5 days before freezing at −80°C (RT-OMBUF).

To investigate the impact of method choice for sample collection on the overall microbiome diversity and composition, fecal material of participant samples (PSs) and negative bacterial controls (NCB) were used. Preservation of fecal material at room temperature (RT) was tested under three conditions, tubes with Zymo Research buffer (ZYBUF), OMNIgene·GUT buffer (OMBUF), and a control sample without buffer (ZYCON and OMCON), and compared to sample storage under standardly frozen conditions (−80) (Figure 2).

**Figure 3:**
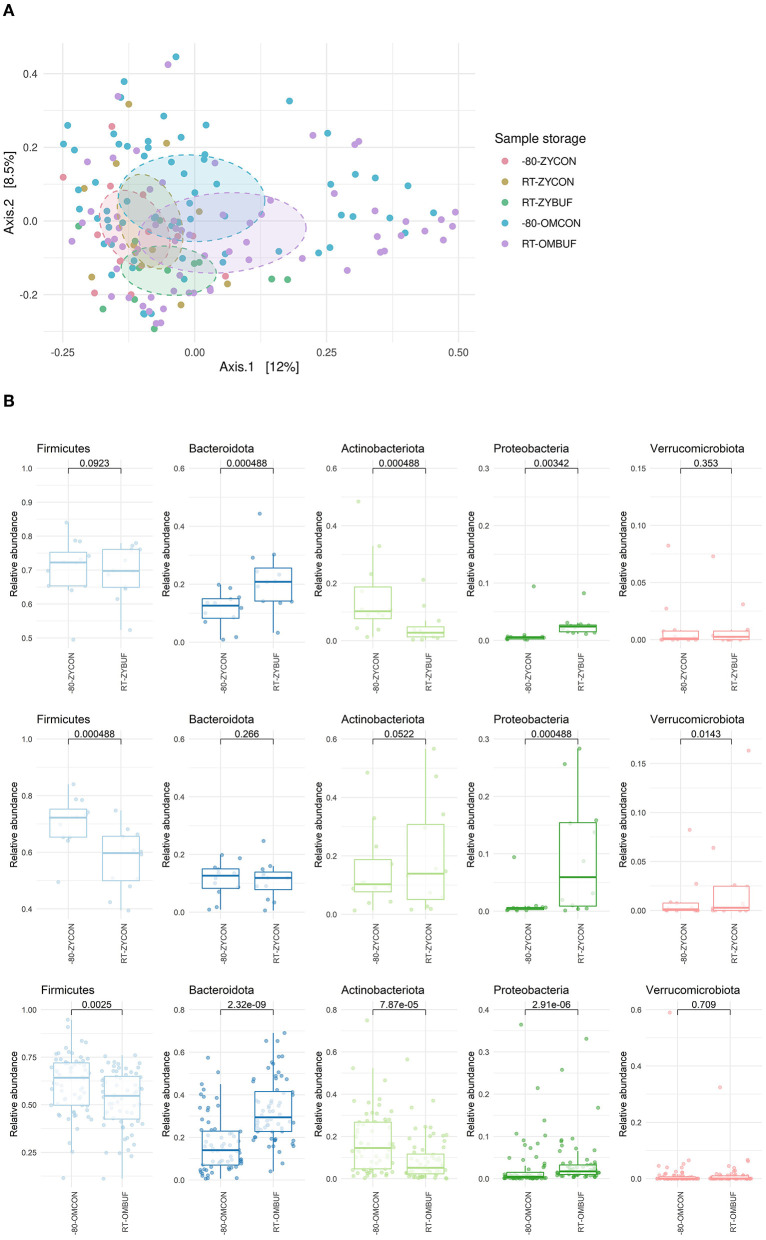
**(A)** Beta-diversity on sample collection and storage. Bray–Curtis distance in a PCoA ordination showed a limited effect on sample collection, only significant for samples stored in the OMNIgene·GUT tubes. *PERMANOVA* testing was performed to test the difference between the two studies (*R*^2^ = 0.026; *p*-adjusted = 0.001), and the type of stabilization buffer compared to the frozen control: OMNIgene·GUT tubes (*R*^2^ = 0.020; *p* = 0.002) and Zymo research tubes (*R*^2^ = 0.046; *p*-adjusted = 1 for RT-ZYBUF, *R*^2^ = 0.032; *p*-adjusted = 1, for RT-ZYCON). **(B)** Boxplots show the relative abundance of the five top most abundant phyla. The differences between the storage conditions were tested with the Wilcoxon test.

No differences between the different collections and storage conditions were observed in alpha-diversity measures, as calculated using the Shannon index, Observed taxa, and Simpson's indices (Supplementary Figure 1a). Next, we looked at the overall differences in microbial composition by using the Bray–Curtis distance in a PCoA ordination (Figure 3A). The largest differences were driven by the two different study groups, PIENTER3 and the *Z*-test study (*R*^2^ = 0.026; *p* = 0.001). Therefore, to control for this confounding effect, comparisons were performed within the aliquots of the same participant in each study. Although there was no significant impact on the microbial composition by sample collection in the Zymo Research tubes, a significant effect was observed when the OMNIgene·GUT tubes were used (*R*^2^ = 0.020, *p* = 0.002). We further investigated the microbial composition at the phylum level (Figure 3B, Supplementary Figure 1b). Sample storage at RT without buffer (RT-ZYCON) influences the microbiome with a higher proportion of Proteobacteria (*p* = 0.000488) and Verrucomicrobiota (*p* = 0.0143) and a lower abundance of Firmicutes (*p* = 0.000488). Samples in the stabilization buffer showed an overrepresentation of Bacteroidota (RT-ZYBUF *p* = 0.000488, RT-OMBUF *p* = 2.32E-09), Proteobacteria (RT-ZYBUF *p* = 0.00342, RT-OMBUF *p* = 2.91E-06), and underrepresentation of Actinobacteriota (RT-ZYBUF *p* = 0.000488, RT-OMBUF *p* = 7.87E-05) compared to standard stored samples. Furthermore, a lower proportion of Firmicutes was observed in samples collected using the OMNIgene·GUT collection tubes (*p* = 0.0025) when compared to the standard frozen condition.

**Figure 4:**
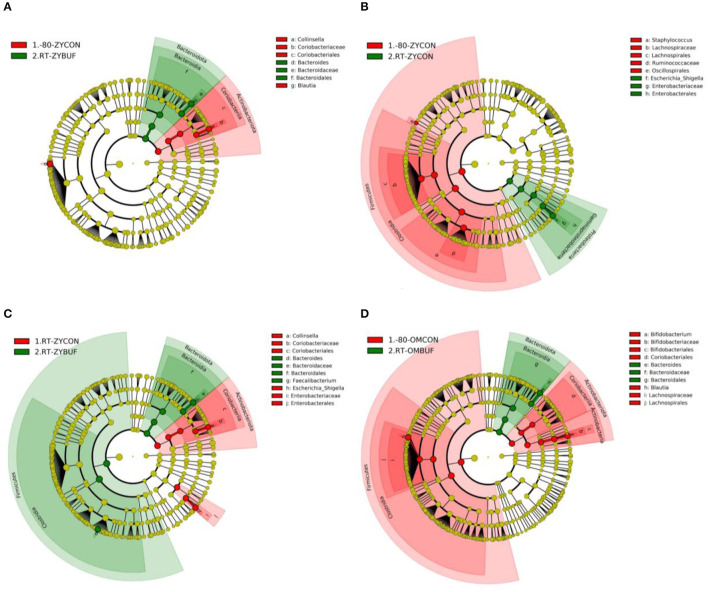
LEfSe analysis showing the significant distinguishing taxa between the different storage methods based on an LDA score >4.0. Results are shown in cladograms, showing the effect of storage at RT, with or without stabilization buffer (RT-ZYBUF, RT-ZYCON, RT-OMBUF) in green, compared to the control samples (−80-ZYCON, −80-OMCON, RT-ZYCON) in red. **(A–D)** The Cladograms show the taxonomic levels represented by rings, with the phylum level in the outermost ring, and the genus level in the innermost ring. Each green or red circle represents significantly different taxa associated with one of the compared groups.

Specific taxa associated with each storage method were identified using the linear discriminant analysis effect size (LEfSe) (Supplementary Figures 2, 4). The taxa that are more abundant in the frozen conditions are represented in red, (−80-ZYCON,-80-OMCON) and in green, those taxa found more abundant when compared to samples stored with or without stabilization buffers at RT (Figures 4A–C for comparisons with RT-ZYBUF, RT-ZYCON, and RT-OMBUF, respectively; 4D for comparison with RT-OMBUF and -80-OMCON). We observed a higher amount of the *Escherichia–Shigella* group (LDA = 4.63) in samples stored at RT without stabilization buffer when compared to the frozen samples, which was not observed in storage with stabilization buffer. These findings were confirmed by comparing relative abundances (Supplementary Figure 3), where we only found a significant difference of Enterobacteriaceae in storage without stabilization buffer (*p*-adjusted = 0.018). As previously observed (Jones et al., 2021), with the RT-OMBUF several taxa within the Firmicutes and Actinobacteriota were less abundant, including *Blautia* (LDA = 4.33) and *Bifidobacterium* (LDA = 4.51), while taxa from the Bacteroidota, such as *Bacteroides vulgatus* (LDA = 4.07), was present in higher abundance (Figure 4A). For the RT-ZYBUF, similar differences were observed, although the effect was less pronounced than that observed with the RT-OMBUF (Figure 4C). Supplementary Table 1 provides an overview of the top 20 most differentially abundant genera. These results confirmed the overgrowth of *Escherichia–Shigella* in samples stored at RT (LDA = 4.6, *p* = 0.013). However, we also observed a higher proportion of the Proteobacteria *Sutterella* in both Zymo buffer (LDA = 3.6, *p* = 0.0021) and OMNIgene·GUT buffer (LDA = 3.5, *p* = 0.000000002).

### Impact of nucleic acid extraction method on the microbial composition

**Figure 5:**
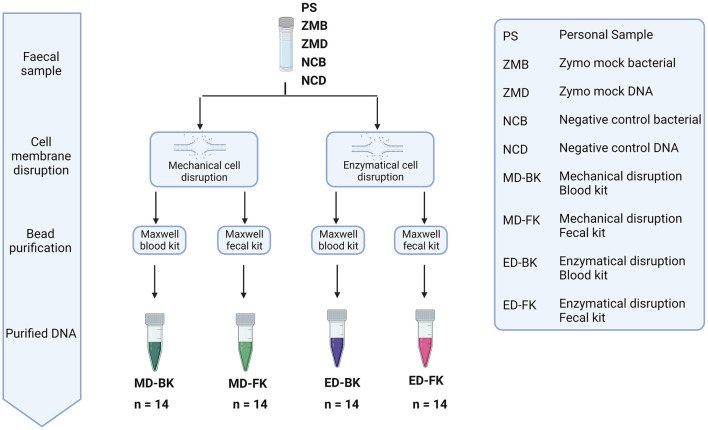
Overview of the different methods for DNA extraction. Personal samples of five donors were stored under two conditions, one aliquot was directly frozen (−80-ZYCON), and the other aliquot of the sample was stored at RT for 3–5 days in Zymo research collection tubes (RT-ZYBUF). These fecal samples, together with two positive and two negative controls were used to test the effect of mechanical or enzymatical cell disruption. Furthermore, we looked into the difference in DNA purification using the Maxwell^®^ RSC Whole Blood DNA Kit and the Maxwell^®^ RSC Fecal Microbiome DNA Kit.

To investigate the effect of the different extraction methods on the fecal microbiome composition and diversity, the personal material of five different donors, a mock community sample, and negative control were used (Figure 5). Two aliquots of the personal samples of five different donors were stored: one control sample stored under standard storage conditions (−80-ZYCON) and an additional aliquot was stored in the presence of Zymo Research stabilization buffer for 3–5 days prior to freezing at −80°C (RT-ZYBUF). The efficiency of each extraction method was determined by absolute quantification of the total DNA yield using the Quantus Fluorometer and the bacterial yield using a universal 16S rRNA gene qPCR.

**Figure 6:**
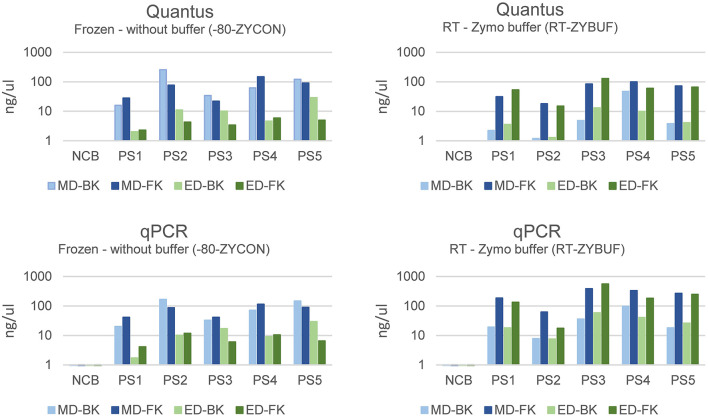
The bacterial yield of samples using mechanical (MD) or enzymatical disruption (ED) and purified with the Maxwell RSC Blood DNA kit (BK) or the Maxwell RSC Fecal Microbiome DNA kit (FK). The DNA concentration was measured using the Quantus Fluorometer and the bacterial DNA using a universal 16S rRNA gene qPCR and represented in ng/μl.

Samples stored without stabilization buffer were extracted more efficiently with mechanical disruption (MD, mean = 79.51, stdev = 49.37) when compared to enzymatical lysis (ED, mean = 9.67, stdev = 7.91) (Figure 6). Interestingly, this effect was not observed in samples stored with stabilization buffer. For the latter, the best performing method of those tested regarding total bacterial yield, was purification with the Maxwell RSC Fecal Microbiome DNA kit (FK) when compared to the Maxwell RSC Blood DNA kit (BK), regardless of how the samples were disrupted.

**Figure 7:**
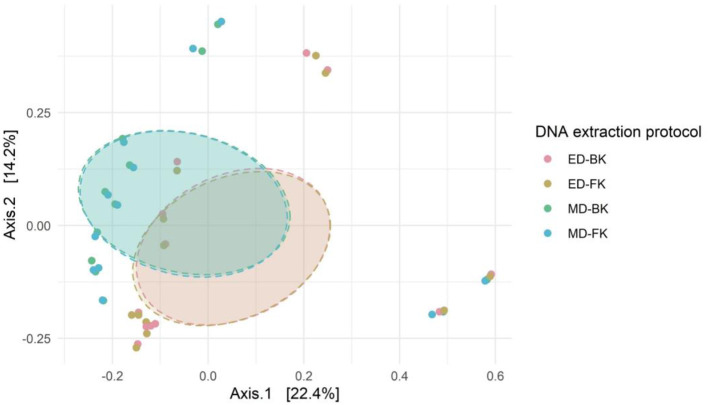
Bray–Curtis distance in a PCoA ordination shows the difference in overall microbial community structure of the different groups (Bead-beating using the Blood and Fecal kits, i.e., MD-BK and MD-FK, and similarly for lysis buffer, i.e., ED-BK and ED-FK). PERMANOVA testing for the method for cell disruption (*R*^2^ = 0.08585; *p*-adjusted = 0.001) and purification kit (*R*^2^ = 0.00016; *p*-adjusted = 1).

Alpha- and beta-diversity analyses were performed on both fecal samples and positive controls for each DNA extraction method tested (*n* = 12; five donor samples in standard tubes, five donor samples in Zymo buffer, and two positive Zymo mock controls). There were no significant differences in alpha-diversity measures between the different extraction methods (Supplementary Figure 4). On the overall community structure, significant differences were observed between the different methods for cell disruption; however, the choice of DNA purification kit showed no effect on the microbial community (Figure 7).

**Figure 8:**
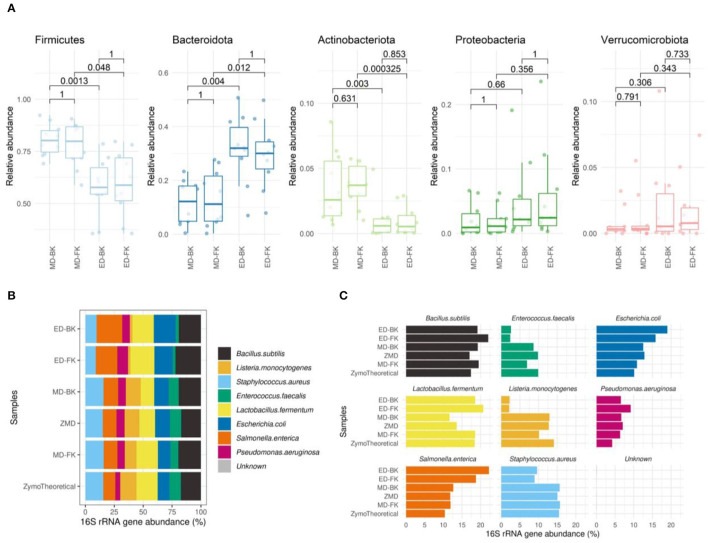
**(A)** Boxplots show the relative abundance of the top most abundant phyla. The Wilcoxon test was used to calculate the adjusted *p*-values of the differences between the DNA extraction methods. **(B)** Spearman's correlation of the Mock samples extracted by the different methods, compared to the theoretical composition of the Mock community sample (MD-FK rho = 0.933, ZMD rho = 0.9, MD-BK rho = 0.75, ED-FK rho = 0.633, ED-BK rho = 0.517). **(C)** Barplots of eight bacterial strains included in the Zymo mock sample.

At the phylum level, no differences were observed between the different DNA purification kits tested (Figure 8A). In contrast, the method for cell disruption influenced the microbial composition significantly. Samples that were enzymatically lysed showed an underrepresentation of Firmicute*s* and Actinobacteriota and an overrepresentation of Bacteroidota when compared to the mechanically lysed cells.

When investigating the mock community sample, by using Spearman's correlation to the theoretical composition provided by the manufacturer [ds1706_zymobiomics_microbial_community_standards_data_sheet.pdf (zymoresearch.com)], we observed the highest correlation in those samples disrupted with bead-beating and lysates purified using the Maxwell RSC Fecal Microbiome DNA kit (rho = 0.933) (Figure 8B). The lowest correlation was found in the enzymatically lysed samples, mostly driven by the underrepresentation of the theoretical *Enterococcus faecalis* and *Listeria monocytogenes* (Figure 8C).

### Limited effect of library preparation on the overall community structure

**Figure 9:**
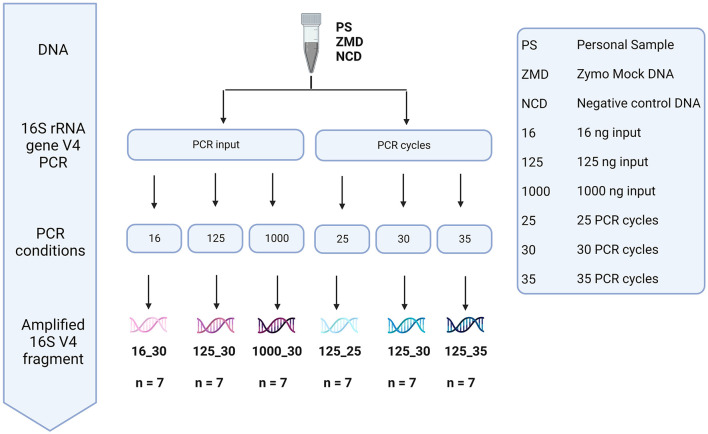
Overview of the different methods tested during library preparation. The effect of bacterial DNA input (16, 125, and 1,000 pg) and PCR cycles (25, 30, and 35 cycles) was tested using the DNA of three participant samples, three negative controls, and one positive Zymo mock control.

To assess the effect of differences in protocols during the amplification of the V4 region of the 16S rRNA gene, DNA material of three different donors, three negative controls, and one positive (Zymo mock) control were used (Figure 9). We analyzed the impact of bacterial DNA input (using 16, 125, and 1,000 pg) and PCR cycles (25, 30, and 35 cycles).

**Figure 10:**
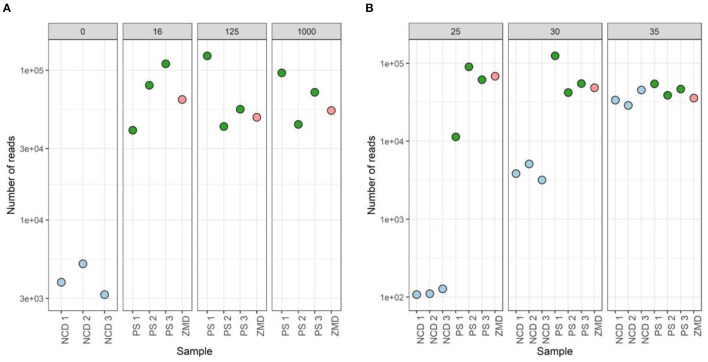
Sequenced reads of three participant samples (PSs), three negative controls DNA (NCD), and one positive control, Zymo mock DNA (ZMD). The effect of different bacterial inputs **(A)** and PCR cycles **(B)** during the 16S rRNA gene V4 region PCR on the number of reads sequenced.

None of the tested conditions resulted in significant differences in both alpha- and beta-diversity measures (Supplementary Figure 5). However, analysis of the sequencing depth showed that, while the different conditions did not influence the number of reads in the donor samples (mean = 64,437, stdev = 30,753), these had an impact on the negative controls (included during the DNA extraction step). A higher number of PCR cycles resulted in a higher amount of contaminant reads in the negative extraction controls (average number of reads detected in negative controls of 116, 4,045, and 36,044 for 25, 30, and 35 cycles, respectively). For 35 cycles, the number of reads detected in negative controls was even comparable to those observed for true samples (Figure 10).

To identify the contaminant reads introduced in the different steps, we used the decontam package. Prevalence-based identification detected 24 contaminant ASVs when analyzing all negative extraction controls (Supplementary Table 2). Negative extraction controls (NCD) prepared with 35 cycles during amplification showed the highest number of reads for all (24/24 taxa detected, average number of reads 14,450), where *Delftia* showed to be the main contaminating genus. When using 30 cycles, we observed a reduction of read counts for all contaminants (23/24 taxa detected, average number of reads 1,459). Samples prepared with 25 cycles showed the lowest number of contaminating sequences (9/24 taxa, average number of reads 45). Contaminants from the Comamonadaceae, *Ralstonia*, and *Mesorhizobium* genera were also found in donor 1 (using 30 and 35 cycles) and a mock community sample (using 30 cycles). None of the contaminant sequences were identified in the true samples when amplified with 25 PCR cycles.

**Figure 11:**
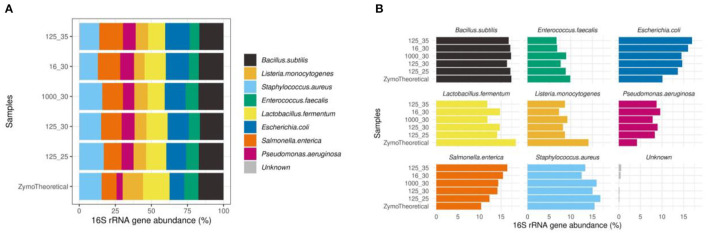
**(A)** Effect of PCR conditions on mock communities during library preparation compared to the theoretical composition using a Spearman's correlation. **(B)** Barplots of eight bacterial strains included in the Zymo mock sample.

Furthermore, we observed that the mock community sample showed the highest correlation to the theoretical composition when using 125 pg input DNA and 25 PCR cycles (rho = 0.833). In mock communities, changes in the number of PCR cycles have a greater impact on the microbial composition than the DNA input, mostly driven by a higher proportion of *Escherichia coli* and *Salmonella enterica* (Figure 11).

### Inter-run variation during sequencing

**Figure 12:**
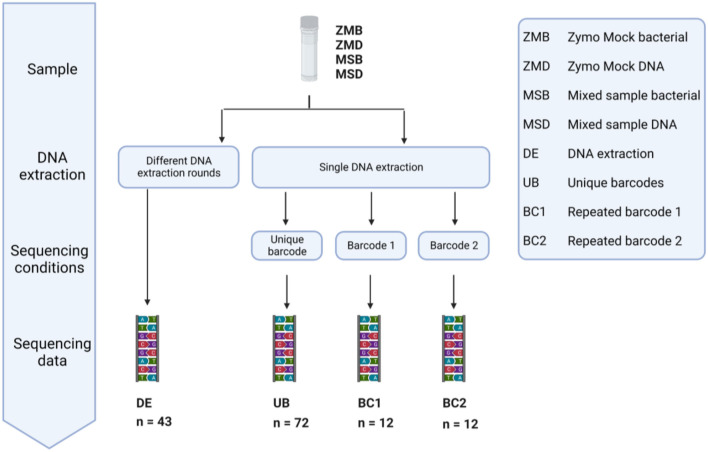
Overview of the conditions tested to measure the variation in large microbiome studies introduced during different DNA extraction rounds, multiple sequencing runs, and the use of barcodes as a unique identifier. DNA of 20 Zymo mock community samples and 23 mixed samples was extracted in different DNA extraction (DE) rounds. The effect of sequencing with a different barcode was tested by repeated sequencing of Zymo mock DNA and Mixed sample DNA 36 times using a different Illumina barcode (UB). The same DNA samples (ZMD and MSD) were sequenced six times using the same Illumina barcode (BC1 and BC2).

In our study, we included 20 Zymo mock bacterial (ZMB) communities and 23 mixed bacterial samples (MSB) to investigate the impact of different DNA extraction rounds. In addition, we used 48 Zymo mock DNA (ZMD) and 48 mixed sample DNA (MSD) to identify the bias introduced during sequencing in different runs with unique barcodes or the same barcode (Figure 12). These samples were classified into four different groups: fecal samples extracted in different DNA extraction (DE) rounds, DNA samples amplified with unique barcodes (UBs), and DNA samples amplified with repeated barcodes (BC1 and BC2).

**Figure 13:**
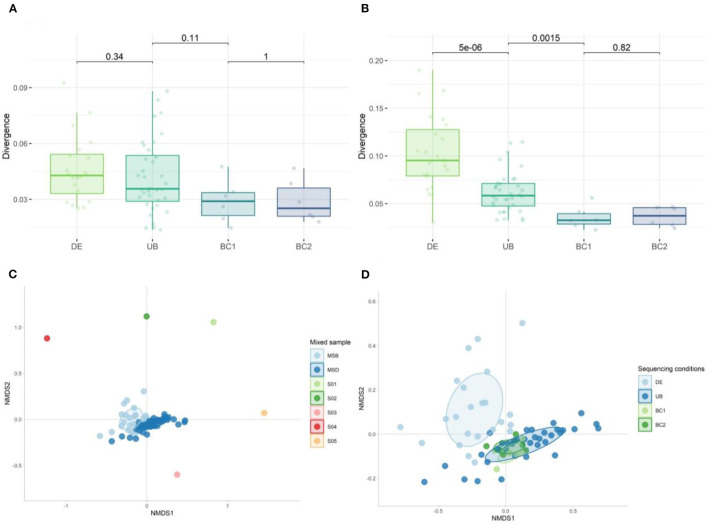
Divergence of **(A)** mock samples and **(B)** mixed samples when comparing the effect of DNA extraction rounds and the use of different barcodes on the heterogeneity within the set of samples. The Wilcoxon test was used to calculate the adjusted *p*-values between all tested conditions. **(C)** Non-metric multidimensional scaling (NMDS) plot of the five donor samples (S01–S05) used to generate the mixed sample used as a positive control in DNA extraction (DE *n* = 23) and sequencing runs (UB *n* = 36, BC1 *n* = 6, and BC2 *n* = 6) (*n* = 71). **(D)** NMDS plot of the 30 mixed samples sequenced: fecal samples extracted in different DNA extraction (DE) rounds, DNA samples amplified with unique barcodes (UBs), and DNA samples amplified with the same barcodes (BC1 and BC2).

We calculated the group divergence, a measure to quantify microbiota heterogeneity within a given sample set with respect to a reference, using the Bray–Curtis dissimilarity method. Although none of the conditions tested on the mock samples were significantly different, sequencing with the same barcodes (BC1 and BC2) showed the lowest divergence (Figure 13A). Significant differences in adjusted *p*-values (Figure 13B) were observed when looking at the more complex mixed samples, showing the largest heterogeneity in the samples extracted in different DNA extraction (DE) rounds compared to the samples extracted in one run (UB). A significantly higher divergence was also found in samples sequenced with different barcodes (UB) when compared to those using the same barcode (BC1 or BC2) (Figure 13B).

We analyzed the overall bacterial communities using a non-metric multidimensional scaling (nMDS) approach (Figures 13C, D). In the divergence quantification as well as the nMDS, we observed the largest spread within the DE group, indicating that different rounds of DNA extraction introduced the most variation during sample preparation for sequencing from all the tested conditions. Using different barcodes (UB) during sequencing has a modest effect on the microbial composition when compared to the results obtained with the use of the same barcode (groups BC1 and BC2).

## Discussion

Biases introduced by methodological differences have been shown to influence microbiome profiles (Choo et al., 2015; Lim et al., 2018; Chen et al., 2020), making it difficult to compare results between studies and research groups. To overcome this challenge, numerous studies have been done to establish a standardized way of setting up microbiome studies (Sinha et al., 2015; Knight et al., 2018; Tourlousse et al., 2021), including a comprehensive checklist (STORMS) developed by a multidisciplinary team of researchers, which can be used as a guide toward a concise and complete reporting of microbiome studies (Mirzayi et al., 2021). Despite the importance of these validated protocols, in a field that is continuously evolving, it is essential to keep innovating methods to improve our current standards. Furthermore, with microbiome studies increasing in number and size, study designs and methodology need to be optimized on a larger scale to improve accessibility for underrepresented geographical locations. In this study, we aimed to contribute to the existing knowledge on the impact that different steps during a microbiome study can have on the overall microbiota diversity and composition and to provide more insight into the biases introduced during the entire workflow and how to control for these.

Sample collection using the standard method (i.e., immediately freezing upon sampling and transporting to the laboratory maintaining the cold chain) is not always feasible (Song et al., 2016; Penington et al., 2018; Wang et al., 2018; Chen et al., 2020). Collecting and transporting samples at room temperature, however, promote the overgrowth of facultative anaerobes and the potential degradation of nucleic acids by the breakdown of strict anaerobes. To prevent this, several methods including different stabilization buffers have been developed in recent years (Song et al., 2016; Natarajan et al., 2021a,b; Plauzolles et al., 2022). In line with the results reported by Roesch et al. (2009), we found a higher amount of Enterobacteriaceae in samples stored at RT for 3–5 days, when compared to the same sample when directly frozen. This was largely prevented by the use of the OMNIgene·GUT and the Zymo research DNA stabilization systems. However, we did observe an increase of *Sutterella* in both Zymo research and OMNIgene·GUT preservation systems, an effect already reported by Choo et al. (2015), explaining the increase of Proteobacteria we observed in all storage conditions tested. Although the overgrowth of Enterobacteriaceae was prevented by the use of the stabilization buffer, we did observe substantial changes in other taxa after storage at RT in the Zymo tubes and OMNIgene·GUT tubes. Both stabilization methods resulted in microbiome profiles with lower proportions of Actinobacteriota *(Collinsella, Bifidobacterium)*, a finding that has been reported before in studies comparing the OMNIgene·GUT to other collection methods (Penington et al., 2018; Wang et al., 2018; Jones et al., 2021). Furthermore, we observed an overestimation of Bacteroidota (*Bacteroides*) and an underrepresentation of Firmicutes (*Blautia*) in all stabilization buffers used. The observed differences were comparable between both preservation systems, and although the impact of this collection method appeared larger in the OMNIgene·GUT samples, this could be due to the larger number of samples used to study this storage system.

In our study, DNA extraction had a significant on the microbial composition, where mechanical disruption of the cells has proven to give the most reliable outcomes (Claassen et al., 2013; Kennedy N. A. et al., 2014; Costea et al., 2017). To evaluate a faster, automated method for DNA extraction, we tested enzymatic lysis and compared this to mechanical lysis. As expected from previous literature, we found a higher DNA yield in the bead-beating samples without stabilization buffer, suggesting this to be a more effective way of cell disruption compared to enzymatical lysis. However, this difference in DNA yield was not observed in the fecal samples extracted in the presence of stabilizing buffer (RT-ZYBUF), indicating that Proteinase K treatment is more effective in fecal samples that are previously diluted and homogenized. Although DNA extraction with enzymatical lysis showed a similar yield compared to mechanical lysis, we did observe a significant difference in the compositional profiles. Mock community samples showed the highest correlation of the bead-beating samples when compared to the theoretical composition. Mock samples lysed enzymatically showed an underrepresentation of *Enterococcus faecalis* and *Listeria monocytogenes*, both gram-positive bacteria which are known to be more difficult to break (Sjöberg et al., 2020). In our study to improve reproducibility, we automated the lysate purification after cell disruption by using a Maxwell RSC system (McGaughey et al., 2018), comparing the Maxwell RSC Blood kit (traditionally used) and the newly developed Maxwell RSC Fecal kit. Both methods resulted in very comparable results with modest to no effect on diversity measures and compositional profiles. Although differences are very minor, when compared to the theoretical mock community composition, the Maxwell RSC Fecal kit showed a higher correlation, making this the preferred method for this sample type.

We observed that a higher number of PCR cycles lead to the accumulation of chimera, point mutations, and artifacts, as previously shown (Wu J. Y. et al., 2010; Kennedy K. et al., 2014). Our data confirmed these observations showing an increase in contaminant ASVs in the negative controls using more PCR cycles. However, most of these contaminants were no longer observed in the presence of fecal material or using a positive control (mixed sample or mock community), suggesting that this effect is mainly problematic during the preparation of low-biomass samples (Salter et al., 2014; Hasrat et al., 2021). Overall from our observations, we propose ~125 pg input DNA and 25 PCR cycles as optimal parameters during library preparation for human fecal samples when using the methods evaluated in this study. In cases where 25 cycles are insufficient to obtain enough material (such as low-biomass samples), extensive analyses and the inclusion of negative controls should be exercised.

To track biases introduced in the various steps for library preparation and sequencing within a microbiome study, it is necessary to include repeated, complex, technical controls. Our data showed no significant differences in the heterogeneity measured in the different control groups using the commercially available community standard (ZMB or ZMD). Differences in factors such as GC content or the presence of specific taxa in databases can influence the sequencing results, suggesting that mock communities alone are not sufficient to control for quality in fecal microbiome samples (Dohm et al., 2008; Simon et al., 2019). We observed an effect when using the more complex mixed sample (MSB), although the limitation here is the unknown composition. However, this can serve as a technical replicate with properties similar to the true samples between multiple batches of sample processing. The highest divergence was observed in the DE group (where the impact of DNA extraction was investigated, followed by the UB group, indicating that both DNA extraction and the use of different Illumina barcodes have an impact during library preparation. The use of one unique barcode resulted in a low heterogeneity in the samples, even though these were sequenced in different sequencing runs, indicating that there was no batch effect observed between the different runs.

Observations from our study may be subject to the low number of samples used for some of the analyses, i.e., to investigate the influence of nucleic acid extraction and library preparation on the overall community structure. Furthermore, we are aware that we were not able to test all available methods and protocols, and we limited these to the commonly used approaches in our laboratory. Finally, these results were obtained in the same laboratory, neglecting the effect of different equipment and laboratory environment. Collaborative studies between multiple laboratories are necessary to test the reproducibility and comparability of datasets from different studies. With novel tools continually emerging, future methodological work will always be needed to expand our current knowledge.

Standard guidelines to process microbiome studies will reduce technical variation across multiple studies, which will allow the comparison of results between different research groups worldwide. Effective, accessible standardization will increase the available data to a broad range of diseases, ethnical backgrounds, and geographic locations. Expanding microbiome data is necessary, as human microbiome research nowadays is dominated by research groups in highly developed countries, neglecting most of the world's population (Gupta et al., 2017; He et al., 2018; Abdill et al., 2022). A more global perspective will harness the full potential of the microbiome to use for targeted strategies of prevention, treatment, and maintenance of health.

## Data availability statement

The data presented in the study are deposited in the The European Nucleotide Archive (ENA) repository, under accession number PRJEB59099.

## Ethics statement

The studies involving human participants were reviewed and approved by Medical Ethics Committee Noord-Holland. The patients/participants provided their written informed consent to participate in this study.

## Author contributions

JK and SF designed the study, analyzed, and interpreted the data. JK and LT performed experiments. JK wrote the article with support from SF and SS. SS contributed to the interpretation of the results and critically revised the article. All authors contributed to the article and approved the submitted version.

## References

[B1] Abdill R. J. Adamowicz E. M. Blekhman R. (2022). Public human microbiome data are dominated by highly developed countries. PLoS Biol. 20, e3001536. 10.1371/journal.pbio.3001536 35167588 PMC8846514

[B2] Anand S. S. (2022). chkMocks: An R Package to Compare Mock Community Samples in Microbiome Amplicon Sequencing Studies (0.1.03). Paris: Zenedo.

[B3] Bahl M. I. Bergström A. Licht T. R. (2012). Freezing fecal samples prior to DNA extraction affects the Firmicutes to Bacteroidetes ratio determined by downstream quantitative PCR analysis. FEMS Microbiol. Lett. 329, 193–197. 10.1111/j.1574-6968.2012.02523.x 22325006

[B4] Callahan B. J. McMurdie P. J. Rosen M. J. Han A. W. Johnson A. J. A. Holmes S. P. (2016). DADA2: high-resolution sample inference from Illumina amplicon data. Nat. Methods 13, 581–583. 10.1038/nmeth.3869 27214047 PMC4927377

[B5] Caporaso J. G. Lauber C. L. Walters W. A. Berg-Lyons D. Lozupone C. A. Turnbaugh P. J.. (2011). Global patterns of 16S rRNA diversity at a depth of millions of sequences per sample. Proc. Natl. Acad. Sci. U. S. A. 108, 4516–4522. 10.1073/pnas.1000080107 20534432 PMC3063599

[B6] Chen C. C. Wu W. K. Chang C. M. Panyod S. Lu T. P. Liou J. M.. (2020). Comparison of DNA stabilizers and storage conditions on preserving fecal microbiota profiles. J. Formos Med. Assoc. 119, 1791–1798. 10.1016/j.jfma.2020.01.013 32111519

[B7] Choo J. M. Leong L. E. Rogers G. B. (2015). Sample storage conditions significantly influence faecal microbiome profiles. Sci. Rep. 5, 16350. 10.1038/srep16350 26572876 PMC4648095

[B8] Claassen S. du Toit E. Kaba M. Moodley C. Zar H. J. Nicol M. P. (2013). A comparison of the efficiency of five different commercial DNA extraction kits for extraction of DNA from faecal samples. J. Microbiol. Methods 94, 103–110. 10.1016/j.mimet.2013.05.008 23684993 PMC5809576

[B9] Costea P. I. Zeller G. Sunagawa S. Pelletier E. Alberti A. Levenez F.. (2017). Towards standards for human fecal sample processing in metagenomic studies. Nat. Biotechnol. 35, 1069–1076. 10.1038/nbt.3960 28967887

[B10] Davis N. M. Proctor D. M. Holmes S. P. Relman D. A. Callahan B. J. (2018). Simple statistical identification and removal of contaminant sequences in marker-gene and metagenomics data. Microbiome 6, 226. 10.1186/s40168-018-0605-2 30558668 PMC6298009

[B11] Dohm J. C. Lottaz C. Borodina T. Himmelbauer H. (2008). Substantial biases in ultra-short read data sets from high-throughput DNA sequencing. Nucl. Acids Res. 36, e105. 10.1093/nar/gkn425 18660515 PMC2532726

[B12] Fan Y. Pedersen O. (2021). Gut microbiota in human metabolic health and disease. Nat. Rev. Microbiol. 19, 55–71. 10.1038/s41579-020-0433-9 32887946

[B13] Fouhy F. Deane J. Rea M. C. O'Sullivan Ó. Ross R. P. O'Callaghan G.. (2015). The effects of freezing on faecal microbiota as determined using MiSeq sequencing and culture-based investigations. PLoS ONE 10, e0119355. 10.1371/journal.pone.0119355 25748176 PMC4352061

[B14] Gupta V. K. Paul S. Dutta C. (2017). Geography, ethnicity or subsistence-specific variations in human microbiome composition and diversity. Front. Microbiol. 8, 1162. 10.3389/fmicb.2017.01162 28690602 PMC5481955

[B15] Halfvarson J. Brislawn C. J. Lamendella R. Vázquez-Baeza Y. Walters W. A. Bramer L. M.. (2017). Dynamics of the human gut microbiome in inflammatory bowel disease. Nat. Microbiol. 2, 17004. 10.1038/nmicrobiol.2017.4 28191884 PMC5319707

[B16] Harris V. C. (2018). The significance of the intestinal microbiome for vaccinology: from correlations to therapeutic applications. Drugs 78, 1063–1072. 10.1007/s40265-018-0941-3 29943376 PMC6061423

[B17] Hasrat R. Kool J. de Steenhuijsen Piters W. A. Chu M. L. J. Kuiling S. Groot J. A.. (2021). Benchmarking laboratory processes to characterise low-biomass respiratory microbiota. Sci. Rep. 11, 17148. 10.1038/s41598-021-96556-5 34433845 PMC8387476

[B18] He Y. Wu W. Zheng H. M. Li P. McDonald D. Sheng H. F.. (2018). Regional variation limits applications of healthy gut microbiome reference ranges and disease models. Nat. Med. 24, 1532–1535. 10.1038/s41591-018-0164-x 30150716

[B19] Honda K. Littman D. R. (2016). The microbiota in adaptive immune homeostasis and disease. Nature 535, 75–84. 10.1038/nature18848 27383982

[B20] Johns M. S. Petrelli N. J. (2021). Microbiome and colorectal cancer: a review of the past, present, and future. Surg. Oncol. 37, 101560. 10.1016/j.suronc.2021.101560 33848761

[B21] Jones J. Reinke S. N. Ali A. Palmer D. J. Christophersen C. T. (2021). Fecal sample collection methods and time of day impact microbiome composition and short chain fatty acid concentrations. Sci. Rep. 11, 13964. 10.1038/s41598-021-93031-z 34234185 PMC8263620

[B22] Kennedy K. Hall M. W. Lynch M. D. Moreno-Hagelsieb G. Neufeld J. D. (2014). Evaluating bias of illumina-based bacterial 16S rRNA gene profiles. Appl. Environ. Microbiol. 80, 5717–5722. 10.1128/AEM.01451-14 25002428 PMC4178620

[B23] Kennedy N. A. Walker A. W. Berry S. H. Duncan S. H. Farquarson F. M. Louis P.. (2014). The impact of different DNA extraction kits and laboratories upon the assessment of human gut microbiota composition by 16S rRNA gene sequencing. PLoS ONE 9, e88982. 10.1371/journal.pone.0088982 24586470 PMC3933346

[B24] Knight R. Vrbanac A. Taylor B. C. Aksenov A. Callewaert C. Debelius J.. (2018). Best practices for analysing microbiomes. Nat. Rev. Microbiol. 16, 410–422. 10.1038/s41579-018-0029-9 29795328

[B25] Kozich J. J. Westcott S. L. Baxter N. T. Highlander S. K. Schloss P. D. (2013). Development of a dual-index sequencing strategy and curation pipeline for analyzing amplicon sequence data on the MiSeq Illumina sequencing platform. Appl. Environ. Microbiol. 79, 5112–5120. 10.1128/AEM.01043-13 23793624 PMC3753973

[B26] Lahti L. Shetty S. (2017). Tools for Microbiome Analysis in R Version. Available online at: http://microbiome.github.com/microbiome

[B27] Lim M. Y. Hong S. Kim B. M. Ahn Y. Kim H. J. Nam Y. D. (2020). Changes in microbiome and metabolomic profiles of fecal samples stored with stabilizing solution at room temperature: a pilot study. Sci. Rep. 10, 1789. 10.1038/s41598-020-58719-8 32019987 PMC7000387

[B28] Lim M. Y. Song E. J. Kim S. H. Lee J. Nam Y. D. (2018). Comparison of DNA extraction methods for human gut microbial community profiling. Syst. Appl. Microbiol. 41, 151–157. 10.1016/j.syapm.2017.11.008 29305057

[B29] McGaughey K. D. Yilmaz-Swenson T. Elsayed N. M. Cruz D. A. Rodriguez R. R. Kritzer M. D.. (2018). Comparative evaluation of a new magnetic bead-based DNA extraction method from fecal samples for downstream next-generation 16S rRNA gene sequencing. PLoS ONE 13, e0202858. 10.1371/journal.pone.0202858 30138447 PMC6107275

[B30] McMurdie P. J. Holmes S. (2013). phyloseq: an R package for reproducible interactive analysis and graphics of microbiome census data. PLoS ONE 8, e61217. 10.1371/journal.pone.0061217 23630581 PMC3632530

[B31] Mirzayi C. Renson A. Genomic Standards Consortium Massive Analysis Quality Control Society Furlanello Cesare 31 Sansone Susanna-Assunta 84 Zohra F. Elsafoury S.. (2021). Reporting guidelines for human microbiome research: the STORMS checklist. Nat. Med. 27, 1885–1892. 10.1038/s41591-021-01552-x 34789871 PMC9105086

[B32] Muyzer G. De Waal E. C. Uitterlinden A. (1993). Profiling of complex microbial populations by denaturing gradient gel electrophoresis analysis of polymerase chain reaction-amplified genes coding for 16S rRNA. Appl. Environ. Microbiol. 59, 695–700. 7683183 10.1128/aem.59.3.695-700.1993 PMC202176

[B33] Natarajan A. Han A. Zlitni S. Brooks E. F. Vance S. E. Wolfe M.. (2021a). Standardized preservation, extraction and quantification techniques for detection of fecal SARS-CoV-2 RNA. Nat. Commun. 12, 5753. 10.1038/s41467-021-25576-6 34599164 PMC8486790

[B34] Natarajan A. Han A. Zlitni S. Brooks E. F. Vance S. E. Wolfe M.. (2021b). Standardized and optimized preservation, extraction and quantification techniques for detection of fecal SARS-CoV-2 RNA. medRxiv 48, 17. 10.21203/rs.3.pex-1601/v1 34599164 PMC8486790

[B35] Nearing J. T. Douglas G. M. Hayes M. G. MacDonald J. Desai D. K. Allward N.. (2022). Microbiome differential abundance methods produce different results across 38 datasets. Nat. Commun. 13, 342. 10.1038/s41467-022-28034-z 35039521 PMC8763921

[B36] O'Sullivan D. M. Doyle R. M. Temisak S. Redshaw N. Whale A. S. Logan G.. (2021). An inter-laboratory study to investigate the impact of the bioinformatics component on microbiome analysis using mock communities. Sci. Rep. 11, 10590. 10.1038/s41598-021-89881-2 34012005 PMC8134577

[B37] Penington J. S. Penno M. A. Ngui K. M. Ajami N. J. Roth-Schulze A. J. Wilcox S. A.. (2018). Influence of fecal collection conditions and 16S rRNA gene sequencing at two centers on human gut microbiota analysis. Sci. Rep. 8, 4386. 10.1038/s41598-018-22491-7 29531234 PMC5847573

[B38] Plauzolles A. Toumi E. Bonnet M. Pénaranda G. Bidaut G. Chiche L.. (2022). Human stool preservation impacts taxonomic profiles in 16S metagenomics studies. Front. Cell. Infect. Microbiol. 12, 722886. 10.3389/fcimb.2022.722886 35211421 PMC8860989

[B39] Quast C. Pruesse E. Yilmaz P. Gerken J. Schweer T. Yarza P.. (2013). The SILVA ribosomal RNA gene database project: improved data processing and web-based tools. Nucl. Acids Res. 41, D590–D596. 10.1093/nar/gks1219 23193283 PMC3531112

[B40] R Core Team (2022). R: A Language and Environment for Statistical Computing. R Foundation for Statistical Computing, Vienna, Austria. Available online at: https://www.R-project.org/

[B41] Requena T. Velasco M. (2019). The human microbiome in sickness and in health. Rev. Clin. Esp. 221, 233–240. 10.1016/j.rce.2019.07.004 33998505

[B42] Roesch L. F. Casella G. Simell O. Krischer J. Wasserfall C. H. Schatz D.. (2009). Influence of fecal sample storage on bacterial community diversity. Open Microbiol. J. 3, 40–46. 10.2174/1874285800903010040 19440250 PMC2681173

[B43] Salonen A. Nikkilä J. Jalanka-Tuovinen J. Immonen O. Rajilić-Stojanović M. Kekkonen R. A.. (2010). Comparative analysis of fecal DNA extraction methods with phylogenetic microarray: effective recovery of bacterial and archaeal DNA using mechanical cell lysis. J. Microbiol. Methods 81, 127–134. 10.1016/j.mimet.2010.02.007 20171997

[B44] Salter S. J. Cox M. J. Turek E. M. Calus S. T. Cookson W. O. Moffatt M. F.. (2014). Reagent and laboratory contamination can critically impact sequence-based microbiome analyses. BMC Biol. 12, 87. 10.1186/s12915-014-0087-z 25387460 PMC4228153

[B45] Segata N. Izard J. Waldron L. Gevers D. Miropolsky L. Garrett W. S.. (2011). Metagenomic biomarker discovery and explanation. Genome Biol. 12, R60. 10.1186/gb-2011-12-6-r60 21702898 PMC3218848

[B46] Simon H. Y. Siddle K. J. Park D. J. Sabeti P. C. (2019). Benchmarking metagenomics tools for taxonomic classification. Cell 178, 779–794. 10.1016/j.cell.2019.07.010 31398336 PMC6716367

[B47] Sinha R. Abnet C. C. White O. Knight R. Huttenhower C. (2015). The microbiome quality control project: baseline study design and future directions. Genome Biol. 16, 276. 10.1186/s13059-015-0841-8 26653756 PMC4674991

[B48] Sjöberg F. Nookaew I. Yazdanshenas S. Gio-Batta M. Adlerberth I. Wold A. E. (2020). Are all faecal bacteria detected with equal efficiency? A study using next-generation sequencing and quantitative culture of infants' faecal samples. J. Microbiol. Methods 177, 106018. 10.1016/j.mimet.2020.106018 32795633

[B49] Smith R. J. (2021). School of ecology package for teaching ecological tasks, algorithms and model fitting. J. Biogeogr. 47, 130–142.

[B50] Song S. J. Amir A. Metcalf J. L. Amato K. R. Xu Z. Z. Humphrey G.. (2016). Preservation methods differ in fecal microbiome stability, affecting suitability for field studies. mSystems 1, e00021–e00016. 10.1128/mSystems.00021-16 27822526 PMC5069758

[B51] Szóstak N. Szymanek A. Havránek J. Tomela K. Rakoczy M. Samelak-Czajka A.. (2022). The standardisation of the approach to metagenomic human gut analysis: from sample collection to microbiome profiling. Sci. Rep. 12, 8470. 10.1038/s41598-022-12037-3 35589762 PMC9120454

[B52] Thaiss C. A. Zmora N. Levy M. Elinav E. (2016). The microbiome and innate immunity. Nature 535, 65–74. 10.1038/nature18847 27383981

[B53] Thompson L. R. Sanders J. G. McDonald D. Amir A. Ladau J. Locey K. J.. (2017). A communal catalogue reveals Earth's multiscale microbial diversity. Nature 551, 457–463. 10.1038/nature24621 29088705 PMC6192678

[B54] Tourlousse D. M. Narita K. Miura T. Sakamoto M. Ohashi A. Shiina K.. (2021). Validation and standardization of DNA extraction and library construction methods for metagenomics-based human fecal microbiome measurements. Microbiome 9, 95. 10.1186/s40168-021-01048-3 33910647 PMC8082873

[B55] Turnbaugh P. J. Hamady M. Yatsunenko T. Cantarel B. L. Duncan A. Ley R. E.. (2009). A core gut microbiome in obese and lean twins. Nature 457, 480–484. 10.1038/nature07540 19043404 PMC2677729

[B56] Verberk J. D. M. Vos R. A. Mollema L. van Vliet J. van Weert J. W. M. de Melker H. E.. (2019). Third national biobank for population-based seroprevalence studies in the Netherlands, including the Caribbean Netherlands. BMC Infect. Dis. 19, 470. 10.1186/s12879-019-4019-y 31138148 PMC6537387

[B57] Wang Q. Garrity G. M. Tiedje J. M. Cole J. R. (2007). Naive Bayesian classifier for rapid assignment of rRNA sequences into the new bacterial taxonomy. Appl. Environ. Microbiol. 73, 5261–5267. 10.1128/AEM.00062-07 17586664 PMC1950982

[B58] Wang Z. Zolnik C. P. Qiu Y. Usyk M. Wang T. Strickler H. D.. (2018). Comparison of fecal collection methods for microbiome and metabolomics studies. Front. Cell Infect. Microbiol. 8, 301. 10.3389/fcimb.2018.00301 30234027 PMC6127643

[B59] Wu G. D. Lewis J. D. Hoffmann C. Chen Y. Y. Knight R. Bittinger K.. (2010). Sampling and pyrosequencing methods for characterizing bacterial communities in the human gut using 16S sequence tags. BMC Microbiol. 10, 206. 10.1186/1471-2180-10-206 20673359 PMC2921404

[B60] Wu J. Y. Jiang X. T. Jiang Y. X. Lu S. Y. Zou F. Zhou H. W. (2010). Effects of polymerase, template dilution and cycle number on PCR based 16 S rRNA diversity analysis using the deep sequencing method. BMC Microbiol. 10, 255. 10.1186/1471-2180-10-255 20937143 PMC2964677

[B61] Yang F. Sun J. Luo H. Ren H. Zhou H. Lin Y.. (2020). Assessment of fecal DNA extraction protocols for metagenomic studies. Gigascience 9, giaa071. 10.1093/gigascience/giaa071 32657325 PMC7355182

[B62] Zhao T. Li J. Fu Y. Ye H. Liu X. Li G.. (2020). Influence of gut microbiota on mucosal IgA antibody response to the polio vaccine. NPJ Vacc. 5, 47. 10.1038/s41541-020-0194-5 32566258 PMC7283253

