# Supplementary figures and images for: Reducing bias in microbiome research: Comparing methods from sample collection to sequencing
Source: Front Microbiol. 2023 Mar 30;14:1094800. doi: 10.3389/fmicb.2023.1094800 (PMC10101209; doi:10.3389/fmicb.2023.1094800)

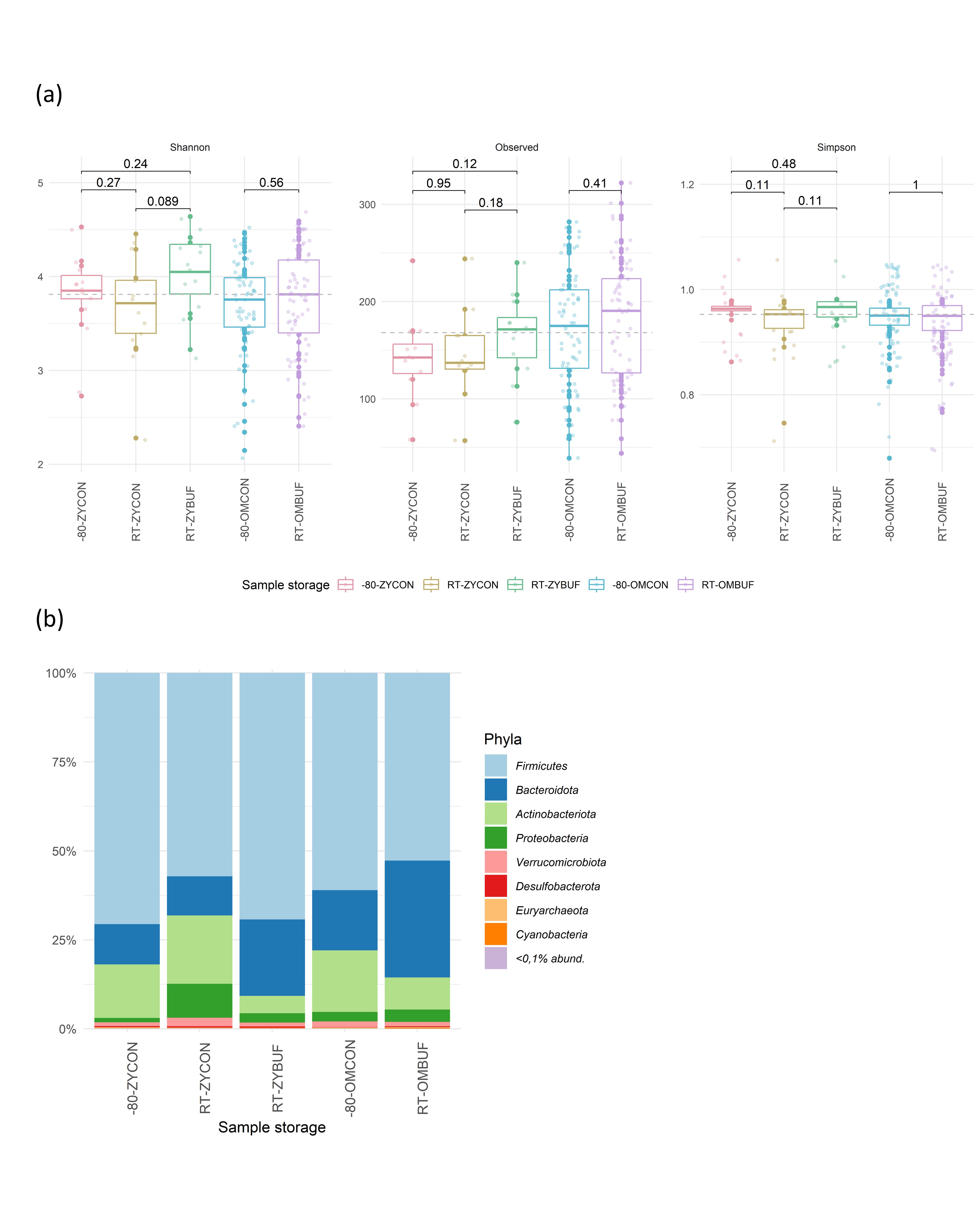

Supplement: Supplementary file 1 [file Image_1.jpg]
