# Supplementary material for: Reducing bias in microbiome research: Comparing methods from sample collection to sequencing
Source: Front Microbiol. 2023 Mar 30;14:1094800. doi: 10.3389/fmicb.2023.1094800 (PMC10101209; doi:10.3389/fmicb.2023.1094800)
Supplement: Supplementary file 2 [file Data_Sheet_1.pdf]

Supplementary materials

Reducing Bias in Microbiome Research: Comparing methods from sample collection to sequencing

Jolanda Kool[1], Liza Tymchenko[1], Sudarshan Shetty[1,2], Susana Fuentes[1]*

1. Centre for Infectious Disease Control, National Institute for Public Health and the Environment (RIVM), the Netherlands.

2. Department of Medical Microbiology and Infection prevention, Virology and Immunology research Group, University Medical Center Groningen, the Netherlands.

*Corresponding author: susana.fuentes@rivm.nl. National Institute for Public Health and the Environment (RIVM), Antonie van Leeuwenhoeklaan 9, 3721 MA Bilthoven, the Netherlands.

Material and methods

Sample selection and study design

In our study, we have used the following definition for technical and biological replicates: **<u>Technical replicates</u>** are repeated measurements of the same sample that represent independent measures of the random noise associated with protocols or equipment. **<u>Biological replicates</u>** are parallel measurements of biologically distinct samples that capture random biological variation, which may itself be a subject of study or a noise source [1].

To this end, we have several sample types in our study. This is also now included as supplementary methods information::

- **Impact of nucleic acid extraction method on the microbial composition:**
    - <u>Biological replicates:</u> In this section we used samples from two different studies, the PIENTER3 and Z-test study. The Zymo Research collection tubes were tested on 12 participants of the Z-test study and the Omnigene gut samples were tested on 65 participants of the PIENTER3 study. Those participants are biological replicates (collected 2 samples in parallel)
    - <u>Technical replicates:</u> All faecal material from those 12 and 65 participants were aliquoted in 2 or 3 to test the storage conditions. Those aliquots are technical replicates. Ideally these replicates should be homogenized before aliquoting, but this was a limitation of the study setup. People were asked to sample at home and store samples immediately after sampling without homogenizing their sample.

- **Impact of nucleic acid extraction method on the microbial composition**
    - <u>Biological replicates:</u> In this section of the manuscript we used 5 individual donors of the Z-test study. These 5 participants collected 3 samples in parallel and were the biological replicates
    - <u>Technical replicates:</u> Faecal material of these 5 participants was homogenized and 4 aliquots of each were used to test all different DNA extraction protocols. In addition to these sample replicates, we extracted DNA from 4 microbial Zymo Mock community samples with all 4 DNA extraction methods. Those Mock community samples are extra technical replicates in our study.

- **Limited effect of library preparation on the overall community structure**
    - <u>Biological replicates:</u> In this part we had 3 biological replicates, DNA of 3 participants from the PIENTER3 study was used to test the effect of library preparation.
    - <u>Technical replicates:</u> The same DNA sample of all participants was used in the 6 library preparation conditions we tested. Furthermore, DNA of the Zymo Mock sample was used as an extra technical replicate to test all conditions.

- **Inter-run variation during sequencing**
    - <u>Biological replicates:</u> For this part we had just 2 biological replicates, a mixed sample of 5 randomly selected participants of the PIENTER3 study and the Zymo Mock community sample
    - <u>Technical replicates:</u> We repeatedly sequenced the mixed sample 71 times and the Zymo Mock community sample 68 times.

Statistical analysis

LEfSe analyses were performed with the online tool Galaxy (harvard.edu) of the Huttenhower lab. LEfSe analysis was performed in 3 steps:

- Step 1: the Kruskall-Wallis test analyzes all features, testing whether the values in different classes are differentially distributed. Features violating the null hypothesis are further analyzed in Step 2.
- Step 2: the pairwise Wilcoxon test checks whether all pairwise comparisons between subclasses within different classes significantly agree with the class level trend.
- Step 3: the resulting subset of vectors is used to build a Linear Discriminant Analysis model from which the relative difference among classes is used to rank the features. The final output thus consists of a list of features that are discriminative with respect to the classes, consistent with the subclass grouping within classes, and ranked according to the effect size with which they differentiate classes.

Results

Effect of sample collection on the overall diversity and composition of the faecal microbiome

(a)

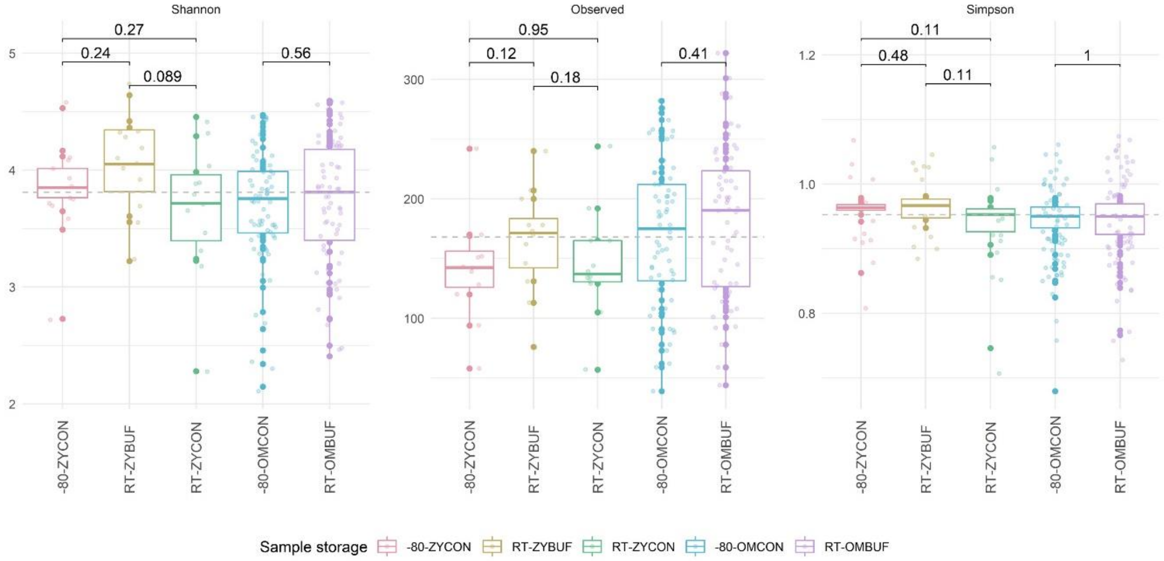

(b)

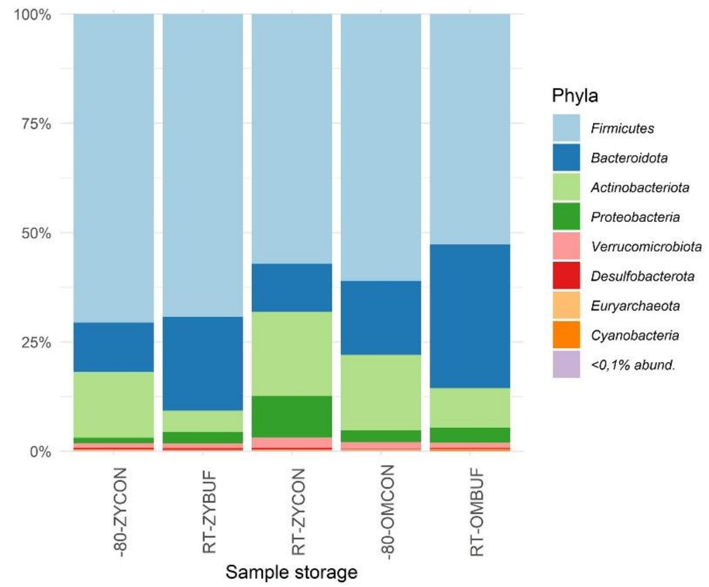

Supplementary figure 1. Comparisons of sample collection and storage methods (a) Alpha diversity indices between different sample collection tubes and storage methods. The Wilcoxon test was used to calculate the adjusted p-values between the different storage methods. None of these differences in alpha diversity were significant.(b) Bacterial composition at the phylum level, stacked bar chart shows the relative abundance of the phyla.

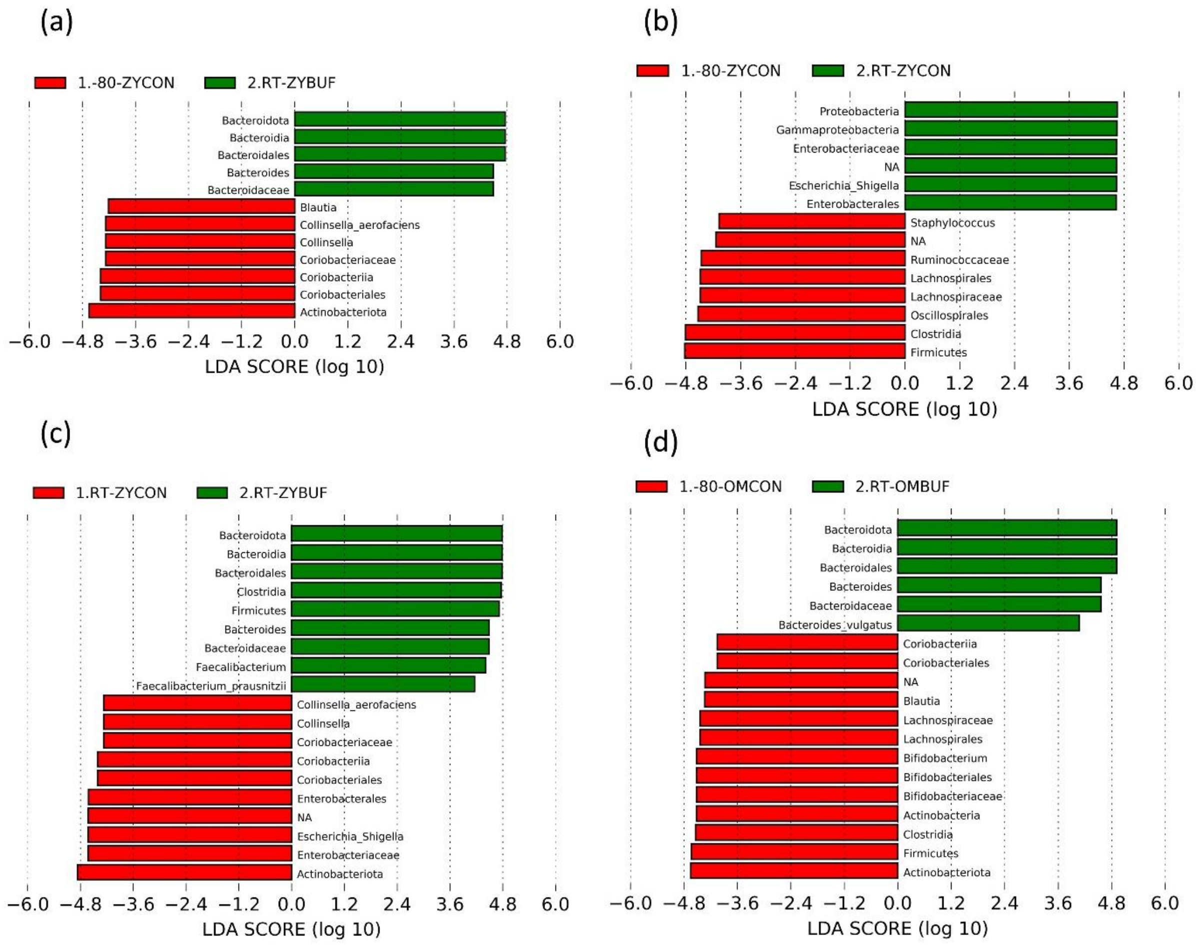

Supplementary figure 2. LEfSe analysis showing the significantly distinguishing taxa between the different storage methods based on an LDA score > 4.0. Results are shown in bar graphs, showing the effect of storage at RT, with or without stabilization buffer ((RT-ZYBUF, RT-ZYCON,RT-OMBUF) in green, compared to the control samples (-80-ZYCON,-80-OMCON, RT-ZYCON) in red.

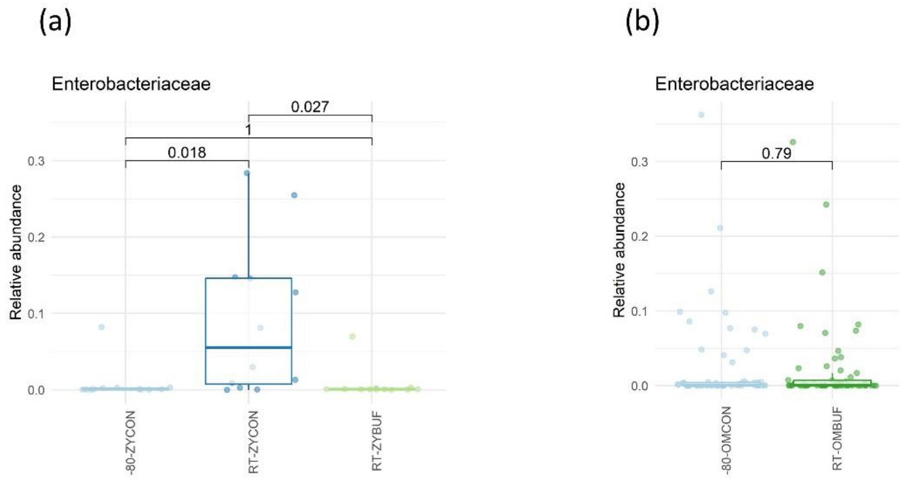

Supplementary figure 3. Boxplots of the relative abundance of Enterobacteriaceae in all storage conditions. Significant differences were calculated with the Wilcox test, when necessary, the p-values were corrected for multiple testing with the Bonferroni correction.

	RT-ZYBUF		RT-ZYCON		RT-OMBUF	
**Taxa**	**LDA**	**p-adjusted**	**LDA**	**p-adjusted**	**LDA**	**p-adjusted**
Bacteroides	4.4	0.051	-3.8	1.000	4.6	0.000
Collinsella	-4.2	0.003	-3.7	1.000	-4.0	0.000
Blautia	-4.2	0.135	-4.1	0.657	-4.3	0.000
Bifidobacterium	-4.4	0.354	4.5	1.000	-4.5	0.003
Escherichia Shigella	-3.5	1.000	4.6	0.036	-3.6	0.714
Fusicatenibacter	-3.5	0.594	-3.7	0.135	-3.6	0.052
Eubacterium hallii group	-3.5	0.180	-3.5	0.342	-3.2	0.148
Sutterella	3.6	0.006	-2.4	0.657	3.5	0.000
Alistipes	3.3	0.639	2.9	1.000	3.5	0.025
Dorea	-3.3	0.534	-3.3	1.000	-3.5	0.001
Parabacteroides	3.4	0.423	3.2	1.000	3.2	0.003
Lachnoclostridium	3.4	0.018	-2.9	1.000	3.2	0.001
Lachnospira	3.4	0.027	3.2	1.000	3.1	0.000
Erysipelotrichaceae UCG 003	-3.6	0.006	3.1	1.000	-3.2	0.245
Eubacterium coprostanoligenes group	-3.6	0.078	-3.3	0.933	-3.1	0.236
Eubacterium eligens group	3.5	0.054	2.7	1.000	3.1	0.118
Lachnospiraceae UCG 004	2.9	0.001	2.4	1.000	2.9	0.000
Lachnospiraceae NK4A136 group	3.5	0.297	-2.8	0.522	3.0	0.013
Odoribacter	2.6	0.693	1.9	1.000	2.8	0.000
Eubacterium xylanophilum group	2.8	0.066	-2.1	1.000	2.4	0.491

Supplementary table 1. LDA score and p-adjusted values of the top 20 most differentially abundant genera, calculated for all conditions compared to the frozen control samples within the same study (RT-ZYBUF and RT-ZYCON compared to -80-ZYCON, RT-OMBUF compared to -80-OMCON). All p-values were corrected for multiple comparisons using the Bonferroni method.

Impact of nucleic acid extraction method on the microbial composition

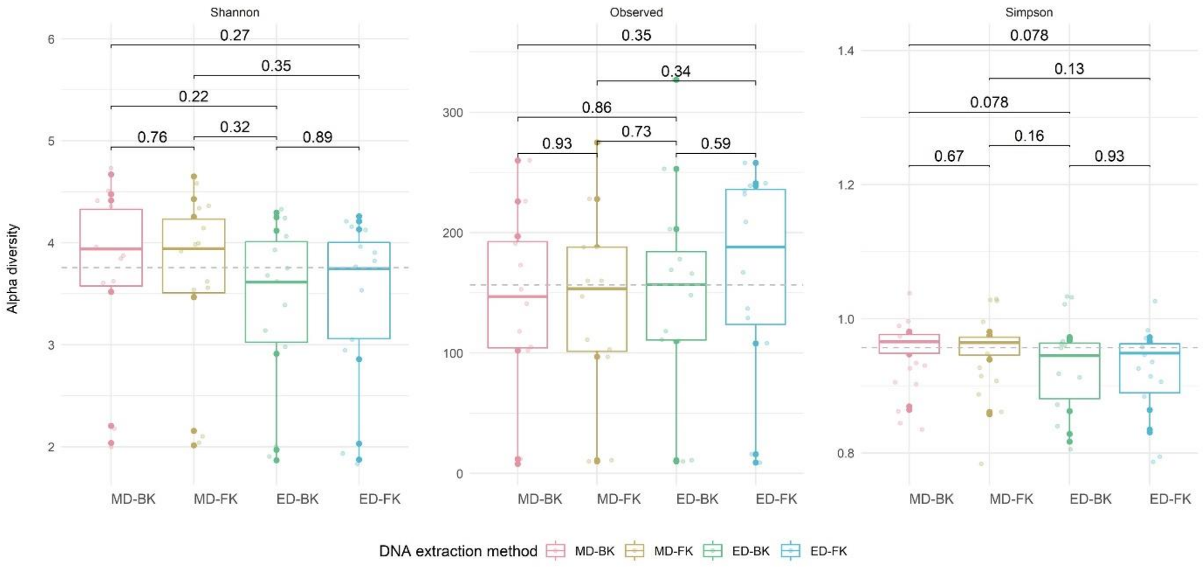

Supplementary figure 4. Alpha and beta diversity measures. Comparison of Shannon index, Observed taxa and Simpson's indices for the different extraction methods. The Wilcoxon test was used to calculate the adjusted p-values between the different DNA extraction methods. There were no significant differences observed in alpha diversity.

Limited effect of library preparation on the overall community structure

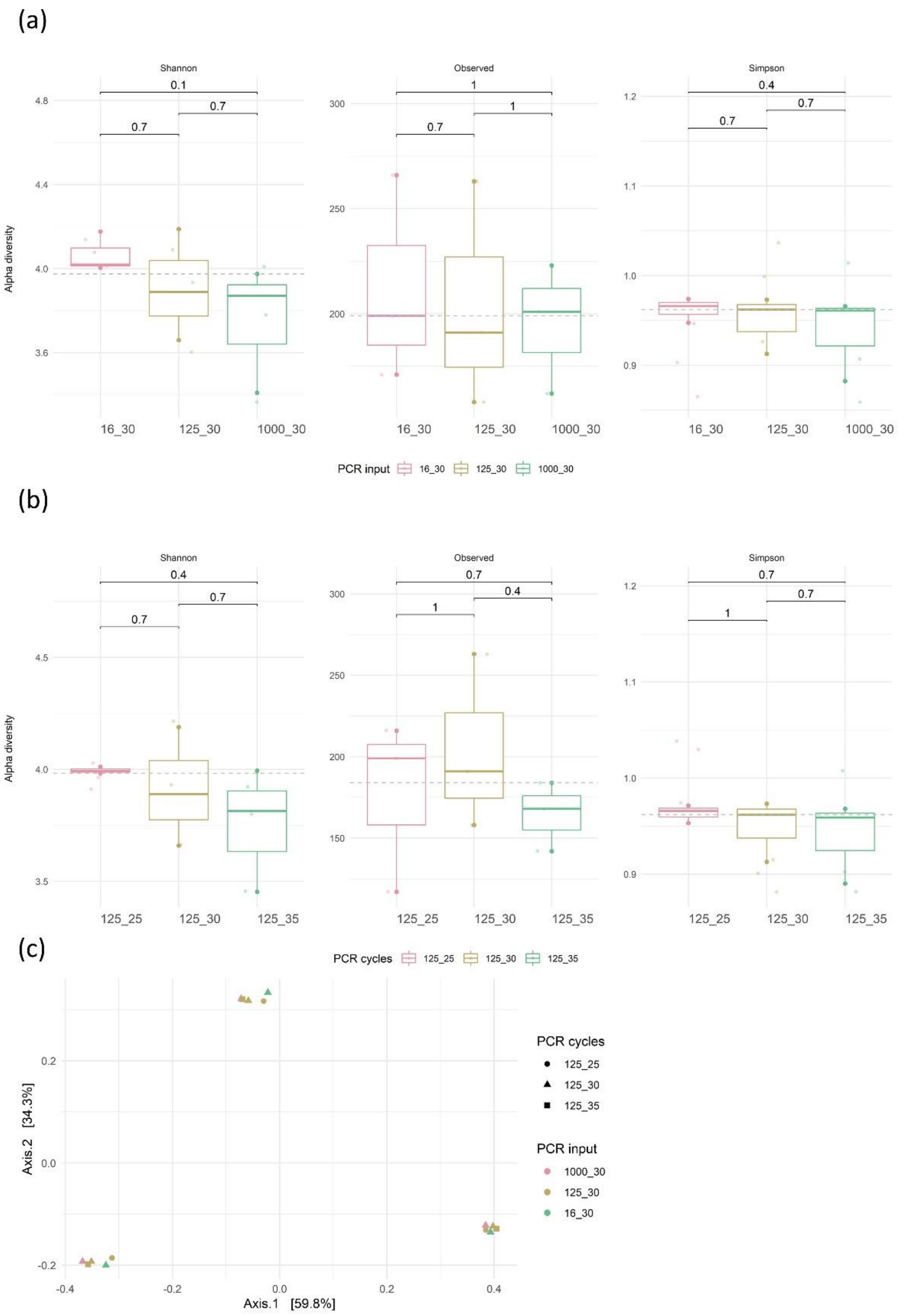

Supplementary figure 5. Comparison of the different conditions tested by using alpha and beta diversity measures. (a) Shannon index, Observed taxa and Simpson's indices were calculated for the different DNA input during amplification of the V4 region of the rRNA 16S gene. Adjusted p-values of the differences between the different PCR conditions were calculated using the Wilcoxon. No

significant difference in alpha diversity was observed. (b) Alpha diversity indexes for the different PCR cycles during V4 amplification. (c) Bray-Curtis distance in a PCoA ordination showing the difference in overall microbial composition of the different groups. perMANOVA results for PCR cycles were (R2= 0.011 ; p-adjusted= 1 for 25 vs 30 cycles, R2= 0.026 ; p-adjusted= 1 for 25 s 35 cycles, R2= 0.004 ; p-adjusted= 1 for 30 vs 35 cycles) and bacterial input (R2= 0.009 ; p-adjusted= 1 for 16 vs 125 pg input, R2= 0.047 ; p-adjusted= 1 for 16 vs 1000 pg input, R2= 0.015 ; p-adjusted= 1 for 125 vs 1000 pg input)

**Contaminants in negative extraction controls (absolute reads)**			
**ASV**	**25 cycles**	**30 cycles**	**35 cycles**
Delftia	27	627	5621
Paenibacillus	6	84	3233
Comamonadaceae	8	245	1829
Massilia	3	77	256
Mesorhizobium		71	875
Ralstonia		83	392
Burkholderia-Caballeronia-Paraburkholderia		57	411
Ralstonia		46	228
Methylobacterium-Methylorubrum		0	205
Hydrogenophilus		19	176
Anoxybacillus		9	156
Ruminococcus	1	33	138
Neisseria		10	159
Meiothermus		4	155
Sphingobacteriales		6	119
Bradyrhizobium		24	113
Kapabacteriales		11	112
Sphingobacteriales		5	84
Lawsonella		11	66
Bacteroides		11	50
Meiothermus		5	28
Corynebacterium		7	23
Brucella		8	11
Micrococcus		6	12
**Sum**	**45**	**1459**	**14450**

Contaminants in participant samples (percentages)			
**ASV**	**25 cycles**	**30 cycles**	**35 cycles**
Comamonadaceae		0.00032	0.00034
Ralstonia		0.00013	0.00014
Ralstonia		0.00003	
Mesorhizobium		0.00007	
**Sum**	**0**	**0.00055**	**0.00048**

Contaminants in positive controls (percentages)			
**ASV**	**25 cycles**	**30 cycles**	**35 cycles**
Comamonadaceae		0.00008	
**Sum**	**0**	**0.00008**	**0**

Supplementary table 2. Table of contaminant ASV[illegible]s detected by the decontam package using the prevalence filtering.

References

1. Blainey, P., M. Krzywinski, and N. Altman, *Points of significance: replication.* Nat Methods, 2014. **11**(9): p. 879-80.
